# Performance Monitoring and Analysis of Task-Based OpenMP

**DOI:** 10.1371/journal.pone.0077742

**Published:** 2013-10-30

**Authors:** Yi Ding, Kai Hu, Kai Wu, Zhenlong Zhao

**Affiliations:** School of Computer Science and Engineering, Beihang University, Beijing, People’s Republic of China; University of Hertfordshire, United Kingdom

## Abstract

OpenMP, a typical shared memory programming paradigm, has been extensively applied in high performance computing community due to the popularity of multicore architectures in recent years. The most significant feature of the OpenMP 3.0 specification is the introduction of the task constructs to express parallelism at a much finer level of detail. This feature, however, has posed new challenges for performance monitoring and analysis. In particular, task creation is separated from its execution, causing the traditional monitoring methods to be ineffective. This paper presents a mechanism to monitor task-based OpenMP programs with interposition and proposes two demonstration graphs for performance analysis as well. The results of two experiments are discussed to evaluate the overhead of monitoring mechanism and to verify the effects of demonstration graphs using the BOTS benchmarks.

## Introduction

Nowadays, multicore CPU design has been widely adopted in current supercomputers and the performance of these systems depends on not only the processor frequency, but also the number of cores. Therefore, how to effectively manage concurrency and contention among cores has become a crucial question for performance improvement.

OpenMP, an API regarded as the de facto standard for multithreaded shared-memory programming, is well suited for current multicore architecture, providing both directives of OpenMP constructs such as parallel regions, sections, and single, etc., and API functions to parallelize the regular structures in version 2.5 [1]. However, the irregular and dynamic structures like recursive routines widely used in current real programs are not well supported in version 2.5 of OpenMP. Therefore, the conception of task is proposed in version 3.0 [2], which allows irregular and independent task constructs to be executed in parallel so as to increase flexibility and efficiency. The task construct in this version is represented by two separate entities: one for task creation and the other for task execution. These entities could be performed in two different dimensions: time and space.

The introduction of the task constructs in OpenMP 3.0 has posed challenges for performance monitoring and analysis, which is still an indispensable means to tune and optimize parallel applications as well as make full use of supercomputers. One major challenge is in order to accurately analyze the programs, we must modify the traditional monitoring and analysis pattern to adapt to the special execution behavior of the tasks. To resolve the challenge, we propose a new method in this study to monitor and analyze task-based OpenMP applications with tied mode, addressing the question how performance tools can include this new dimension (task) in OpenMP paradigm and provide all necessary data to performance analysts so that the application performance could be optimized.

The rest of this paper is organized as follows. Related work is examined in section 2 and the problems of monitoring and analysis in task-based OpenMP program are analyzed in section 3. A proposed solution to the problems identified is described in the following section. In section 5, two experiments are analyzed to validate this solution’s efficiency and effectiveness, which is followed by a conclusion of this study and an outline of future work.

### Related Work

Unlike MPI with PMPI interface, OpenMP specification does not offer a performance profiling standard. To date, three data acquiring methods have been proposed and applied in previous work.

The first method is statistical sampling. The tools subgroup of the OpenMP ARB (Architecture Review Board) has agreed on publishing a runtime API based on this method for profiling applications, as proposed by Sun (Oracle) [3]. Though encouraged to follow this specification, few OpenMP compiler vendors have implemented it in their compilers (with the exception of Sun and Intel [4]). [5] extends this proposal and implements a prototype tool named Sun (Oracle) Studio Performance Analyzer to support tasks. The main shortage of this method is its lack of accuracy to obtain related information.

Another method is built upon direct instrumentation. POMP is the first interface and OPARI [6] as its prototype implementation has been commonly used in performance tools, such as TAU [7] and Scalasca [8]. However, tasks are not currently supported for these tools. The initial work on task instrumentation presented by [9] is simple, without task-identification. [4] proposes a mechanism to identify task instance via extension of OPARI (called OPARI2) and implements it in Score-P [10]. However, its code modification and recompilation are found to be time-consuming and complex because OPARI is a source-to-source instrumentation tool.

Interposition is the third solution to intercept performance data. Although Extrae supports monitoring task-related information for its new version 2.3 [11], the method is still too simple to identify the task instances and their relationships.

With respect to task-based performance visualization analysis, most of the work fails to clearly describe the tasks’ execution behaviors and the relationships among task instances. [5] and [9] just display task execution and waiting time in a coarse grain, while [11] and [12] do not reveal the relationships among task instances. Though [10] maintains the tasks with detailed profiling information, and the tasks in the visualization are independent of main program in the call tree, the dependency relationships among the task instances cannot be clearly presented.

### Problem Analysis

This work intends to offer a promising new approach to monitor and analyze task-based OpenMP paradigm and to overcome the weaknesses mentioned in the above section.

The introduction of the task constructs is the most visible feature of OpenMP 3.0, which adds an additional concurrency dimension for work execution and shifts the paradigm from being thread-centric in nature to task-centric. The thread-centric mode has little control over the work distribution across threads, which could easily cause a problem of load imbalance and has difficulties in processing irregular and dynamic structures. The task in task-centric mode, however, can be constructed in any form of computation, and it is not bound to any particular thread. The scheduling here indicates the assignment of the tasks to OpenMP threads. Their reasonable match would greatly improve the execution efficiency [13]. Using the new concept of task, all the work units are defined as tasks, which include implicit (traditional work units) and explicit ones. In addition, explicit tasks could also be divided into two types: tied and untied tasks [14]. Our approach in this work is limited to tied tasks, mainly because most implementations of untied tasks are immature. At present, the famous GCC compiler and libgomp library for example have not implemented the mode of untied tasks.

Interposition [15] is an effective means to collect performance-related information without source, special compilation or linking. It is defined by the process of placing a new or different library function between the application and its reference to a library function. In real implementation, the library function in the middle, also called wrapper function, is often designed to wrap the real function.

Our goal is to obtain useful task-related performance data with a monitoring library composed of all the wrapper functions and to represent the data in a reasonable pattern to identify performance problems. Three problems are required to be solved owing to the complexity of task-based OpenMP paradigm.

1. Identification of the behavior of task regions, especially of explicit tasks. As an additional dimension of OpenMP programming paradigm, these tasks display different execution behavior. Once created, the OpenMP runtime system will face two choices: it could be either executed immediately or queued for later execution (decided by scheduling policies or related directives). The tracing of task instances is not straightforward, since it integrates all the related information such as their relationships.
2. Integration of explicit tasks with traditional implicit ones. The different behavior of the two types entails different monitoring methods. However, as a whole system, their nomenclature, related information, etc, should be consistent and mutually compatible.
3. Reasonable description of task related information. The two dimensions of the threads and the tasks are required to be displayed in one graph to represent the execution behavior, which is a necessary step for further performance analysis.

## Methods

This section describes a tasked-based monitoring mechanism with the technique of interposition, and introduces two demonstration graphs to reveal, from different perspectives, the relationships among task instances hosted on related threads.

### Task-based Monitoring Mechanism

OpenMP ARP releases only the specification of OpenMP but not the implementation in detail; thus, different compilers have their own specific instantiations. A generalized abstract model is necessary to describe the task execution and monitoring control flows. Then the implementation approaches of the monitoring mechanism, based on a typical compiler, can be studied as a demonstration.

In addition, based on the technology of interposition, the monitoring library (wrapper library) between application layer and OpenMP runtime library is transparent to the user. The runtime library is not allowed to be changed, and some internal data structures cannot be accessed directly either. These factors should be considered in construction of the description model.

The description model with six components is defined as follows.

#### Definition 4.1

(*Task Instance*) *Task Instance*, represented as *taskIns*, is an instance of the basic execution unit in the program, presenting similar behavior to the conception of task in the specification of OpenMP 3.0. It is composed of a series of attributes and behavior depicted below.

- *static attribute*. It is used to describe the inherent characters of *taskIns*, being illustrated as “*taskIns*.ID = xxx”. The common attributes are listed in Table 1.
- *execution behavior*. It is used to describe the operations of *taskIns* in its life-time, being illustrated as “*taskIns*.execute()”. The operations are listed in Table 2.
- *execution state*. It is one of the *static attributes*, represented as “*taskIns*.state = xxx”. The common states are listed in Table 3.

**Table 1:** The list of *static attributes.*

Name	Description
ID	It is used to identify current task instance, being required to present the execution level and position on the form
parentID	It is used to identify the task instance which generates current one
nature	It is used to indicate the nature of current *taskIns* (implicit or explicit)
threadID	It is used to identify the thread which executes current *taskIns*
scheduMode	It is used to indicate whether current *taskIns* is required to immediately execute by the restricted conditions
state	It is used to describe the execution state of current *taskIns* (see Table 3)
data	It represents the required data and context for task execution

**Table 2:** The list of *execution behavior.*

Name	Description
execute	It indicates the execution operation of current *taskIns*
create	It indicates the operation of creating new *taskIns*
synchronize	It indicates the operation of synchronization for current *taskIns*
resume	It indicates the operation of resuming for current *taskIns* as it is suspended before

**Table 3:** The list of *execution states.*

State	Description
EMERING	It describes the state of the generation of current *taskIns*, being abbreviated to EMER
WAITING	It describes the waiting state of current *taskIns*, which is placed into *Task Pool* before execution, being abbreviated to WAIT
RUNNING	It describes the executing state of current *taskIns*, being abbreviated to RUN
SUSPENDING	It describes the suspending state of current *taskIns*, which is caused by the operation of synchronization as well as terminated by the operation of resuming, being abbreviated to PEND
CREATING	It describes the phase of creation operation, being abbreviated to CREAT

#### Definition 4.2

(*Task Pool*) *Task Pool*, is the space organized in a certain pattern to store task instances with related information (including required data, context, and possibly static attributes such as task identifier). Usually, this space is hosted in the memory buffer, and the task instances are organized in the form of linked lists.

#### Definition 4.3

(*Thread Pool*) The term “*Thread*” here indicates the execution unit of OpenMP paradigm (it is provided by the OpenMP runtime system as a user-level thread, not by the operating system as a kernel thread). *Thread Pool* is composed of two layers: thread team and thread. While the implicit task instances bind fixed threads to be executed, the explicit ones are in turn scheduled by *Scheduling Engine* to map the threads in *Thread Pool*.

#### Definition 4.4

(*Scheduling Engine*) *Scheduling Engine*, a user-level scheduling control center mainly aimed at the explicit task instances, monitors the states of *Thread Pool* as well as *Task Pool* online and maps the task instances to threads by the scheduling rules. The concrete scheduling policies depend highly on the implementation mechanism of the special compiler and the runtime library.

#### Definition 4.5

(*Task Control Engine*) It is responsible for the two aspects of preprocessing the task instances: operating the required data and the contexts of these task instances, and assigning the task instances to *Task Pool*.

#### Definition 4.6

(*Execution Pool*) It is an abstract conception for the execution of task instances. Once some task instances enter this pool, the real functions they points to (explicit task instances) or their function entities (implicit task instances) will be executed immediately.

**Figure 1:**
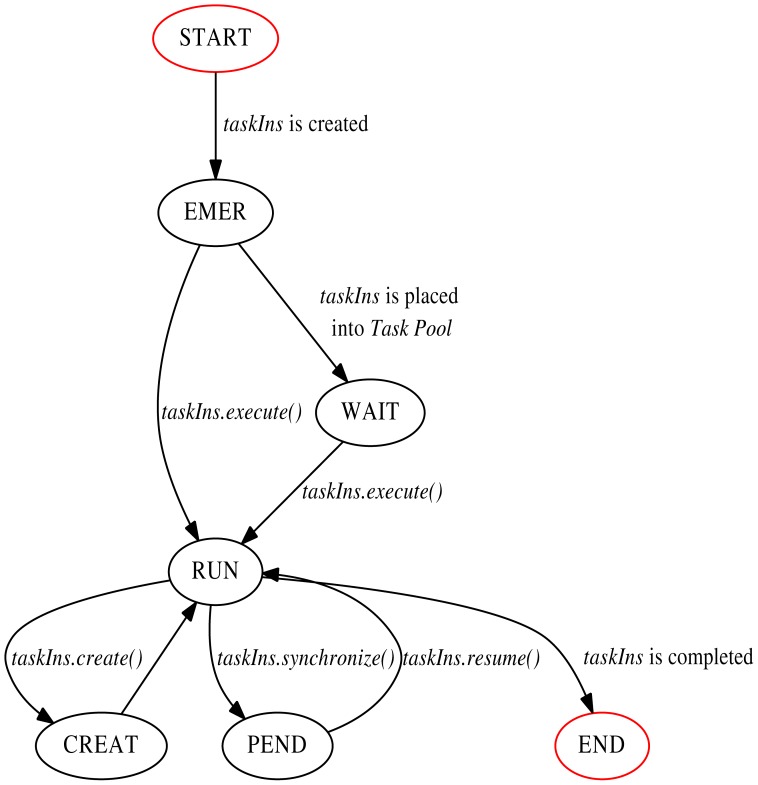
The state transition graph of task execution flow.

**Figure 2:**
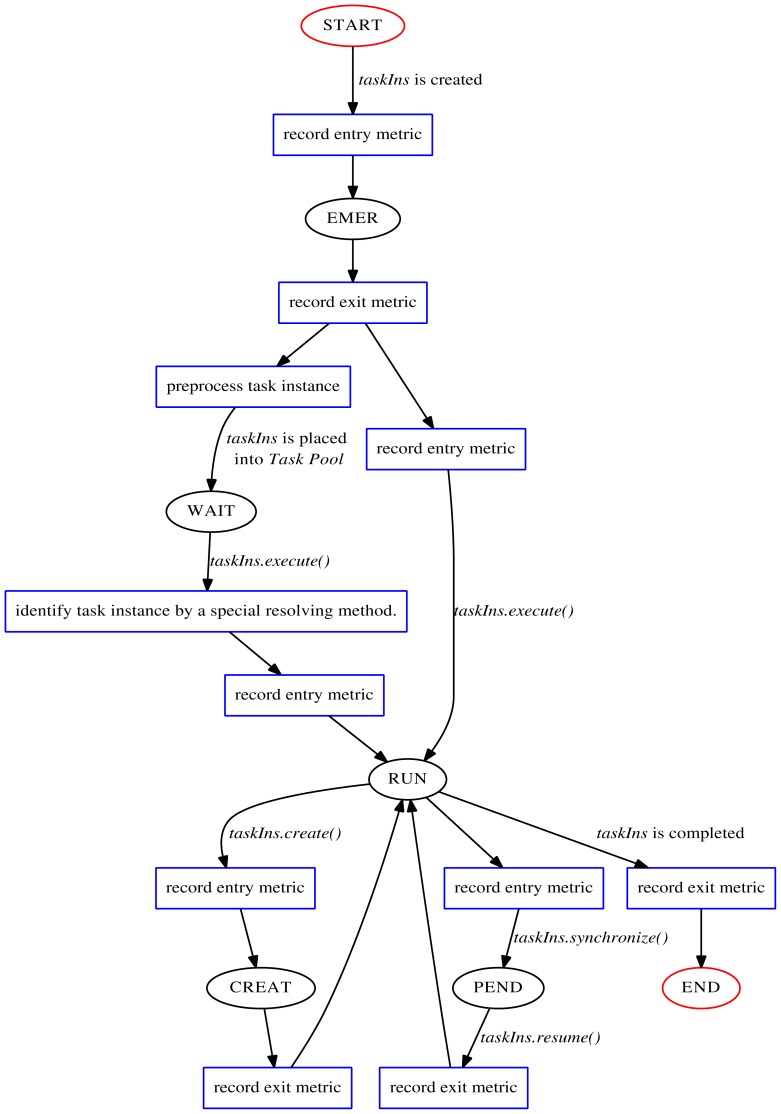
The monitoring principle diagram.

The task execution flow could be described as a state transition graph based on the model described above. It is displayed as Figure 1, starting as the state of “START” and ending at “END” with red circles. In the life-time of a task instance, different contexts, restricted conditions (such as if clause) and execution behavior may cause different state transitions. The key question for performance monitoring using the technique of interposition is to identify the task instance and to record related metrics for each state. The monitoring principle diagram is shown in Figure 2. The monitoring operations, indicated in the blue rectangles at different phases, record related metrics (the most common one is timestamp). Especially, preprocessing *taskIns* before “WAIT” state is to design and store the identification information as well as necessary *static attributes* of the task instance, and in turn to resolve them by special methods after “WAIT” state (before current *taskIns* begins to execute). Then the recording metrics could be mapped to related task instances to reflect correct performance behavior. In addition, our proposal here only support tied tasks for the reason stated above.

As mentioned earlier, a typical implementation of the monitoring mechanism will be studied in the following. Since GNU compiler (GCC) is open source and widely used, its implementation of OpenMP is chosen as our research basis.

**Figure 3:**
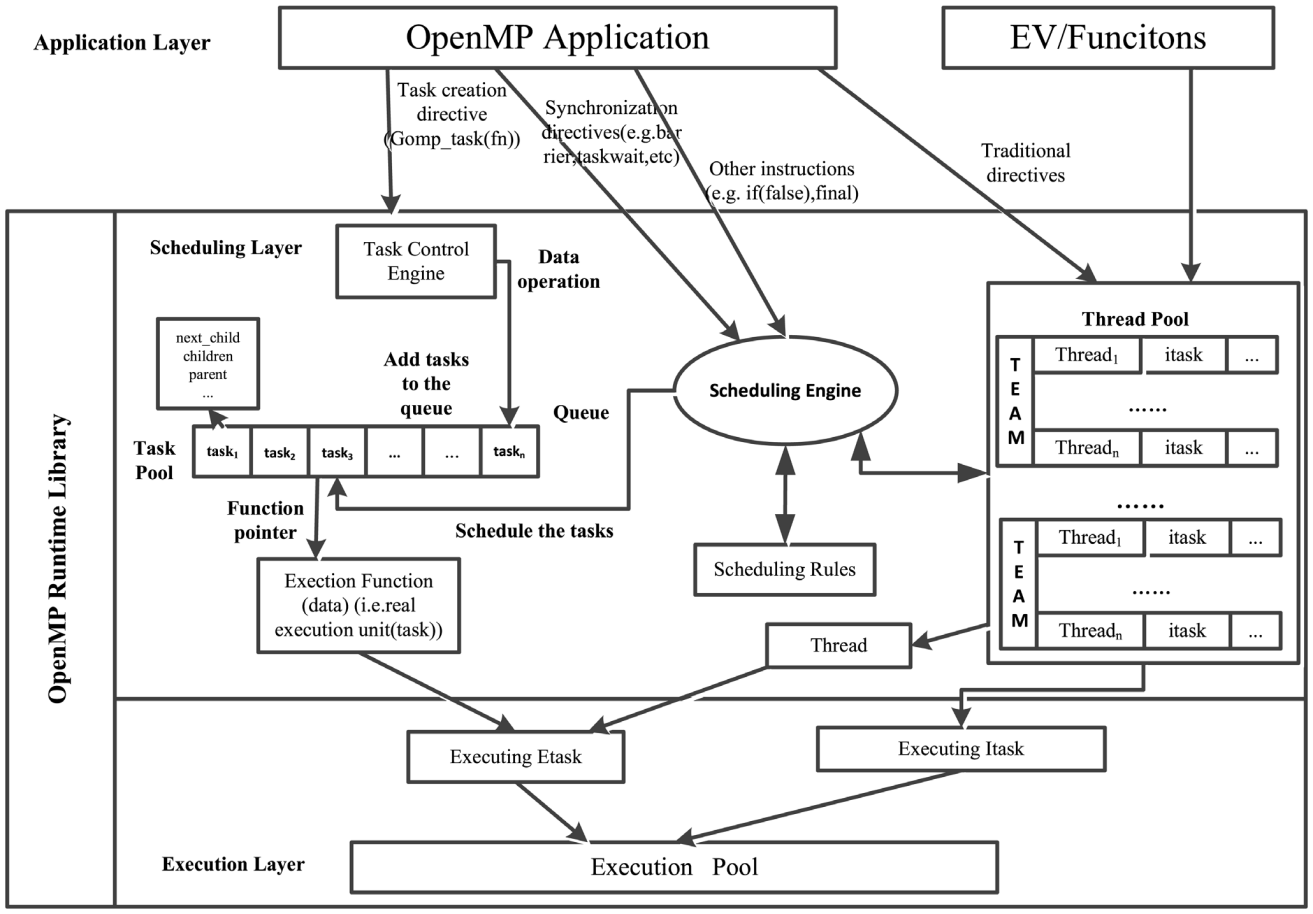
The task execution mechanism of GCC.

We first analyze the task execution mechanism of GCC runtime library (libgomp), shown in Figure 3, and then design a monitoring library. Some abstract terms are used in this figure for a better and simple description. The whole system is divided into three layers: application, scheduling and execution, with the last two involved in OpenMP runtime library. The traditional directives (OpenMP 2.5), environment variables, etc, are independent of *Scheduling Engine*, and corresponding work units (implicit tasks) are binding to a particular thread in one team of *Thread Pool* to be executed immediately. Once created, the explicit task shall enter *Task Control Engine*, which processes related data and makes tasks enqueue into *Task Pool* (in fact, two linked lists are maintained for it). Meanwhile, the scheduling conditions such as synchronization directives, if clause, etc, are accepted by *Scheduling Engine* which also monitors *Thread Pool*. Schedulable tasks, available threads and scheduling rules are combined to decide the order of task execution. Once a task is scheduled, its executive entity will be placed to *Execution Pool* to be performed.

In order to acquire the desired information about the task instances, the monitoring library has to have means to store the task identifier attaching necessary *static attributes*, and resolve it before execution, mapping task instance to related performance data. Two possible ways to implement the monitoring library are discussed in the following part.

**Figure 4:**
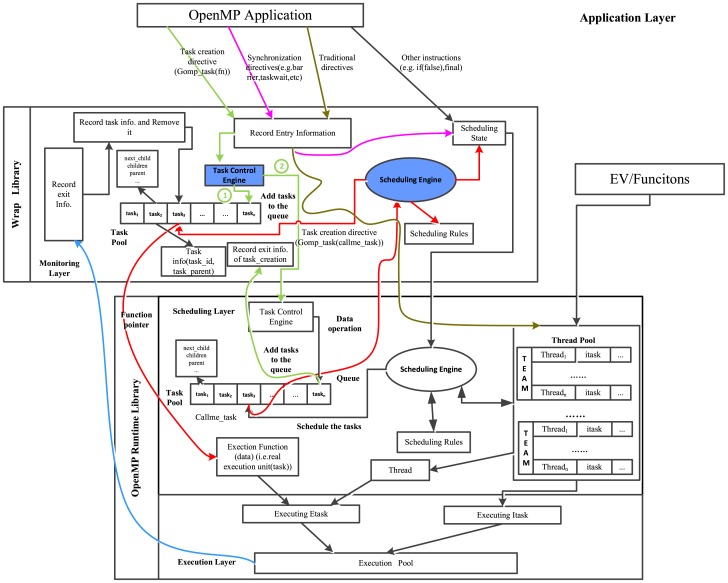
The mechanism of self-scheduling. The flow lines with the same color represent the same execution flow and the circled numbers show execution orders with the same functional module.

Our first scheme described as Figure 4 is to design an independent *Scheduling Engine* similar to the one in OpenMP runtime library. When an explicit task instance is created, *Task Control Engine* in the monitoring library puts it into a *Task Pool* (task enqueue) outside (in the monitoring layer) with its related information and then places it into normal execution flow (the real function pointer is still wrapped). As the real function is invoked in the runtime, the wrapper function is actually executed. Then the monitoring library realizes the task instance and schedules it from the self-maintaining *Task Pool* by the outer *Scheduling Engine* (in the wrap library) according to the rules. This task instance related information is resolved and stored, and then the real function will be called to execute in execution layer. In spite of its accuracy of data acquiring, this proposal causes high overhead because of the overhead-prone locking mechanism used to maintain a *Task Pool* in the monitoring library.

**Figure 5:**
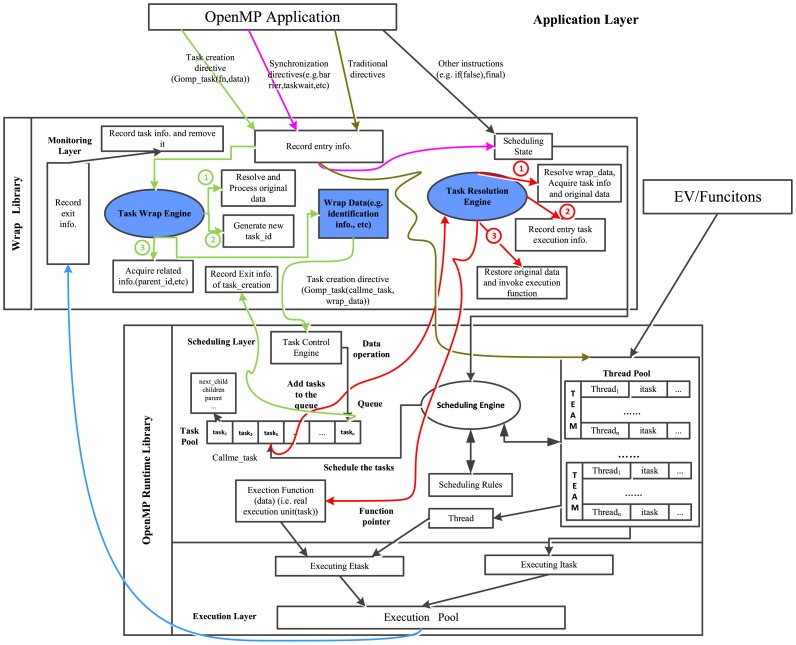
The mechanism of data wrapping. The flow lines with the same color represent the same execution flow and the circled numbers show execution orders with the same functional module. *Task Resolution Engine* and *Task Wrap Engine* are key components in the monitoring library.

To improve efficiency, another proposal is presented as follows. The amount of the key task identification information required to be tracked is limited so that we could attach it to the data region carried by task instance. As scheduled at runtime, the wrapper function intercepts it and resolves the data to record the task instance related information. The original data region is restored as well, with which the real function (executive entity) is invoked to *Execution Pool*. The mechanism is described in detail in Figure 5. The wrapper data is not simply attached. To ensure accuracy, it is necessary to preprocess the data in different situations implicated with the parameters of task creation function.

In addition, the explicit task instance may be suspended in some situations (for example, when the taskwait or barrier directive is encountered). The scheduling mechanism embedded in the runtime library (GUN libgomp) is consistent with the rule of call stack, which is very different from the FIFO scheduler described in [4]. Therefore, the wrapper library could record the performance metrics in this case without extra efforts. The main problem is caused by the separation of task creation and execution.

To integrate explicit task instances with traditional ones, the same information is maintained for them in the monitoring library, especially by consistent naming conventions. OPARI2 utilizes OpenMP thread identifier attaching task count for task identifier definition [4]. However, in OpenMP runtime library, a thread pool is often exploited to reduce the overhead of thread creation and destroying. When a parallel region is encountered, several threads in the thread pool could be activated to execute the program. Then, these threads will be set to sleep until they encounter another parallel construct and several parallel regions could also be constructed in one program with different thread numbers. In this case, the traditional expression is not suitable to be used to represent the unique identifier for the global program with several parallel regions in real meaning. We propose a new nomenclature described as (1)
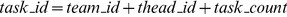
(1)
*team_id* is generated as countering a parallel region, while *thread_id* is the system thread ID which could clearly represent the real execution thread. *task_count* indicates the created task instance with the particular parallel region and system thread. *team_id* and *task_count* are both sequential nature numbers starting from zero. In addition, *+* is the concatenation symbol. *task_id* is unique and globally accessible, which is acquired at the point of task creation and remains unchanged and valid during the lifetime of a task instance.

### Task-based Demonstration Graphs

Classic performance profiles and timeline displays are often in units of functions. The additional parallelization dimension of task in OpenMP paradigm, however, requires new demonstration graphs to clearly present performance behavior. To overcome the limitation of classic visualization pattern, two graphs are introduced and defined as follows.

#### Definition 4.7

(*Task Timeline Graph* (*TTG*)) A graph describes the execution behavior of different threads with respective task instances working on them as time goes by. Different from the traditional timeline graph, the functions are replaced by task-related behaviors and the parent-child relationships are still concatenated in the graph.

#### Definition 4.8

(*Dynamic Task Relationship Graph* (*DTRG*)) A dynamic task relationship graph of OpenMP program *P* with the execution of task instances is defined by a directed flow graph *G = {T, E_p_, E_d_, F}* with a set of nodes *T* and a set of edges *E_p_* as well as *E_d_*. A node *t∈T* represents a task instance executed by a different thread. This execution is still the node’s property which is labeled by a different color (if several task instances execute on the same thread, the respective nodes are also labeled by the same color). An edge *<t_1_, t_2_>∈ E_p_* is a pair of *t_1_, t_2_∈T* where *t_2_* is one child of *t_1_* and *t_1_* is the sole parent of *t_2_*. This edge describes the parent-child relationship between the task instances. Likewise, an edge *<t_3_, t_4_>∈ E_d_* is a pair of *t_3_, t_4_∈T* where *t_4_* is dependent of *t_3_*. This edge describes the dependency relationship between the task instances. The edges belonging to a different set are represented by different shapes in the graph. The first task instance during the *i^th^* parallel region of *P* is defined as *f_i_ ∈F*. If *E_d_* is empty and the number of parallel regions is one, the graph will become a tree structure representing the parent-child relationships among task instances, which is defined as *Dynamic Task Relationship Tree* (*DTRT*).

*DTRG* could accurately describe the task relationships during program lifetime with related profiling information attached. However, *E_d_* still has many redundant edges interwoven in the graph, which will influence the analysis effect and efficiency. To address the problem, two other definitions are then introduced.

#### Definition 4.9

(*Dependency Relationship Path Stack* (*DRPS*)) A dependency relationship path stack is the sequential dependency relationship composed of parts of edges contained in *E_d_* of *DTRG*. It is denoted by *DRPS* = (*t_r1_→t_r2_→t_r3_→ …→ t_rn_*) (*n>2* and *n* is a natural number), starting with a node *t_r1_*, followed by *t_r2_* dependent of *t_r1_*, followed by *t_r3_* dependent of *t_r2_*, and so on. This definition implies that the same node may appear in various *DRPS*. In addition, the dependency relationships in *DRPS* are provided with transitivity (e.g. 

#### Definition 4.10

(*Redundant Dependency Relationship Path* (*RDRP*)) Let *e_dp_ =  <t_rs_, t_re_>* be an edge contained in *E_d_* of *DTRG*, and *DRPS_q_*  =  (*t_r1_* → *t_r2_* → *… t_rn_*) (*n>2* and *n* is a natural number) be one instance of *DRPS*. *e_dp_* is called *Redundant Dependency Relationship Path iff* both of them (*e_dp_* and *DRPS_q_*) satisfy the following conditions: (i) *t_rs_ = t_r1_ and t_re_ = t_rn_*; (ii) *DRPS_q_*⇒*e_dp_ but not the opposite*.

**Figure 6:**
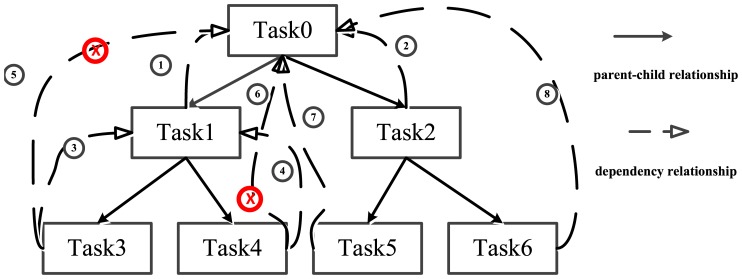
The case of *RDRP* instances. Task0 is the implicit task instance, while the others are explicit ones.


A case is provided here to explain the definitions above, with the core pseudocodes described below. To keep things simple, only one thread is executed in the parallel region. Two task instances are created by the implicit task instance and then they create their own task instances respectively. The *DTRG* of this case is displayed as Figure 6 in which the instances of *RDRP* are labeled with 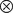 in red color. Task0 is the implicit task instance and its two child task instances are Task1 and Task2. Task1 creates its child task instances: Task3 and Task4, while Task2 has its child task instances of Task5 and Task6. Task0 is dependent of all the descendent task instances because of the implicit barrier directive. In addition, Task1 is dependent of its child task instances due to the taskwait directive. According to the definitions of *DRPS* and *RDRP*, a conclusion is easily drawn. (Task3*→*Task1*→*Task0) and (Task4*→* Task1*→*Task0) are *DRPS* instances, while <Task3, Task0>(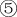),<Task4, Task0>(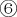) are *RDRP* instances and redundant in this figure.

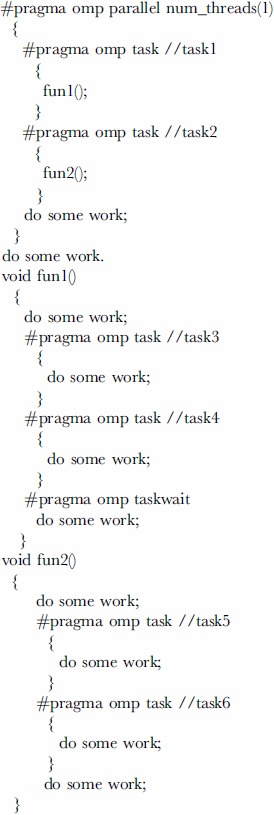


Based on these definitions, a *Redundancy Pruning Method* (*RPM*) is required to efficiently remove *RDRP* in the graph. An obvious method is to go through all the nodes and edges to find the instances of *RDRP*. However, significant costs make this scheme impractical. We thus propose an algorithm based on the feature of GCC, briefly described as **Algorithm 1** (Table 4), which could be easily extended to other implementations and integrated with the generation of *DTRG*. The instances of *RDRP* are mainly generated owing to the synchronization dependency relationships of barrier and taskwait directives in current GNU OpenMP implementation.

**Table 4:** The description of *Redundancy Pruning Method* (*RPM*) (Algorithm 1).

1: **for** each task*i*, *i*∈[*0…n-*1] **do**
2: **if** task*i.bar* is equal to 1 (1 represents it has a barrier property) **then**
3: **for** each descendant of task*i* –task*j* **do**
4: **if** task*j.tw* is equal to 1 (1 represents it has a taskwait property) **then**
5: **if** one edge or *DRPS* exists between task*i* and task*j* **then**
6: **if** one child task of task*j* has one edge to task*i* **then**
7: this edge is one instance of *RDRP* and will be removed;
8: **end if**
9: **end if**
10: **end if**
11: **end for**
12: **end if**
13: **end for**

Algorithm 1: For each task instance, a property of *bar* means whether it has a synchronization point caused by barrier directive, while the property of *tw* means whether it has a synchronization point caused by taskwait directive; *n*: the number of task instances.

In short, *TTG* focuses on task execution flow with the related thread, while *DTRG* on the relationships between the task instances. They depict the task behavior from different perspectives and help identify the performance problems. The real examples will be displayed in the experimental part.

## Experiments and Results

Based on the monitoring mechanism and the demonstration graphs described above, we implemented a prototype monitoring library and some demonstration modules which were integrated into the framework of PAPMAS (Parallel Application Performance Monitoring and Analysis System) [16]–[17]. Two experiments were conducted to evaluate the overhead of performance monitoring and to verify the effect of demonstration graphs with the prototype implementations.

### Experimental Evaluation of Monitoring Overhead

**Figure 7:**
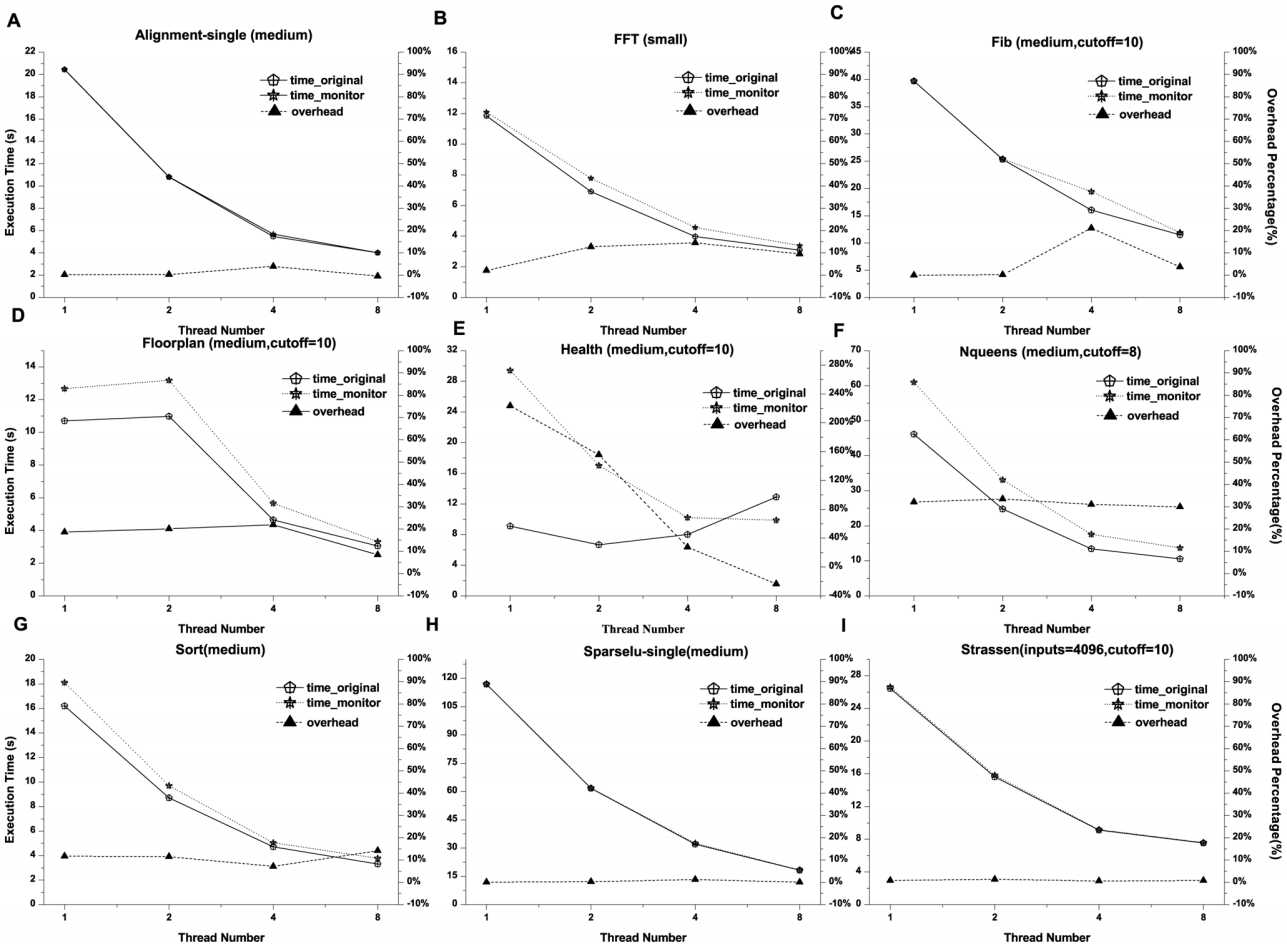
The experimental comparison with monitored and unmonitored version of BOTS benchmarks. The abscissa represents thread number, while the execution time is on the left ordinates, and monitoring overhead in the form of percentage is represented on the right ones. The title means the execution program with its input size and/or cutoff value.

In this part, we monitored the BOTS (Barcelona OpenMP Task Suite) [18] benchmarks and compared execution time with those of the unmonitored version. The experimental platform is an ASUS high performance server (Intel Xeon W3520, 4 cores, 8 threads, 2.67 GHz, 8 G memory capacity, 500 GB hard disk capacity, etc). Nine test programs were compiled with GCC version 4.72. The function of storing performance data was closed for two reasons: first, this part of overhead is necessary for every system and dependent of the storage device; and second, this function is not the focus of this study. Since untied tasks are not supported in our system at present, we evaluated only the tied version of the codes. Otherwise, the cut-off (fib, floorplan, health, nqueens and strassen) and single (alignment and sparselu) versions were chosen if they were provided. The monitored and unmonitored versions were executed with 1, 2, 4, 8 threads respectively and the results are shown in Figure 7. The measurement overheads for alignment, fib, sparselu and strassen are not obvious (less than 10%), while the overheads of fft, floorplan, sort are medium (approximately between 10% and 20%). The program of nqueens was measured with higher overhead (about 30%). Their varied behavior was similar except the result of health was so strange that the overhead gradually tapers with the growth of thread number.

**Figure 8:**
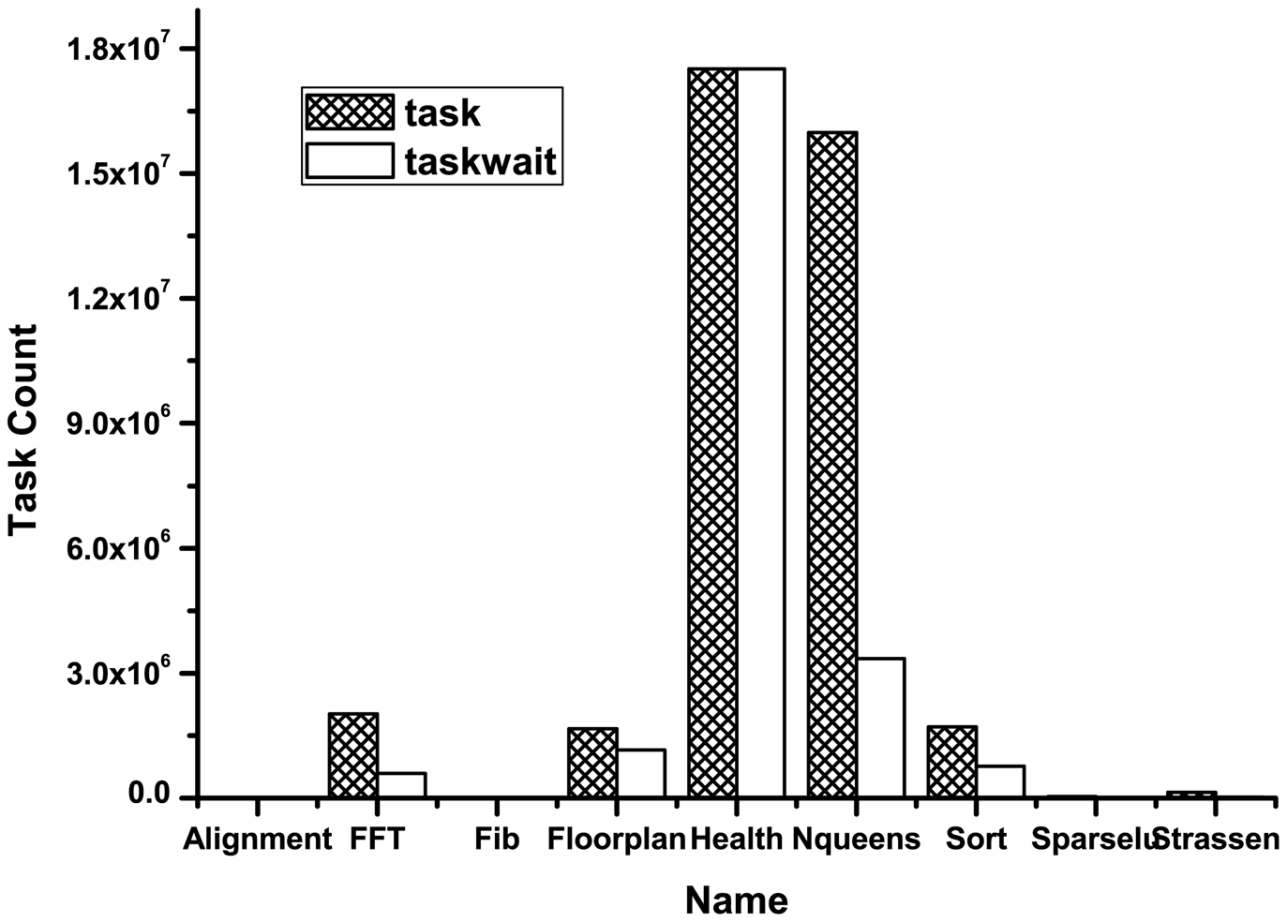
The comparison chart with the counts of task instances and taskwait. The abscissa represents the execution program, while the numbers of task instances and taskwait operation are represented on the ordinate.

As the new dimension introduced, explicit task instances and their related operations are undoubtedly important factors for the overhead. Therefore, another trial was conducted to acquire the number of task instances and the execution times of taskwait (other functions were executed with fixed counts) for all the test codes with sprof tool. The results are displayed in Figure 8. From the graph, it is concluded that there is an approximate linear relationship between the overhead and task count. With a large number of task instances, the overhead of processing and recording performance information could not be ignored. The number of task instance and taskwait for health are both the largest. The lock mechanism is exploited as low level implementation of taskwait, which would increase overhead with growth of thread number owing to the increasing competitions. Since the monitoring library would enlarge task execution time due to the overhead of information processing and recording, the effect of competition lock may be diminished. This explains the phenomena of health.

On the whole, the overhead generated from the monitoring library could be considered acceptable since the measurements provide quite detailed information about the runtime behavior of the application.

### Experimental Verification of Demonstration Graphs

**Figure 9:**
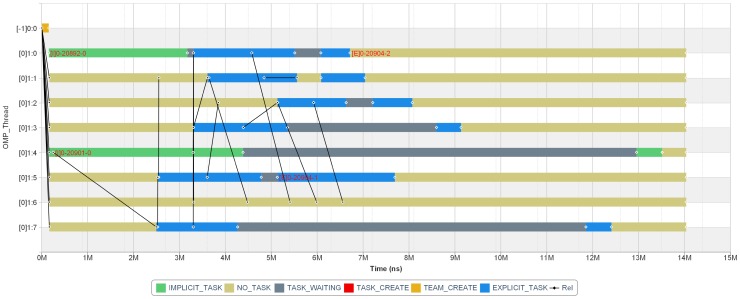
The chart of *TTG*. The abscissa represents the execution time while the ordinate represents the execution threads. The representation [a]b:c on the ordinate indicates parent thread number, level number and current thread number respectively, which could clearly represent the cases of multiple parallel regions and parallel region nesting. The legend explains the used colors related to various task behaviors shown in this chart. TaskID ([E] indicates explicit task instances while [I] represents implicit ones) will be displayed on the rectangular bar in the graph until its length reaches a threshold. In addition, this chart can be scaled for easy viewing.

**Figure 10:**
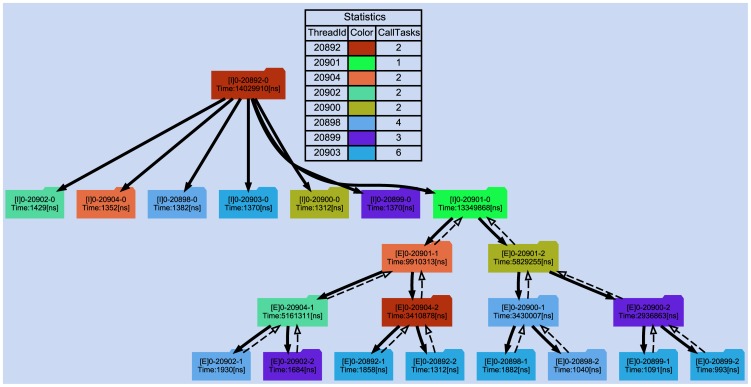
The chart of *DTRG*. The statistics table illustrates the execution threads in the graph with different colors and the number of task instances executed on them. The information attached on the task box is taskID ([E] indicates explicit task instances while [I] represents implicit ones) and execution time.

In this part, a trial was designed for verifying the effect of *TTG* and *DTRG*. Like our first experiment, fib (cut-off = 3, input size is set as “small”) program in BOTS benchmarks was chosen to be monitored and visualized. The two graphs are displayed as Figure 9 and Figure 10 respectively. In *TTG*, the length of abscissa is directly proportional to the time spent in the program. The task behavior with the two dimensions of time and thread is clearly displayed: one thread basically created 2 child task instances, waited for them and then made some summing operations. These steps proceeded recursively from each child task instance until the level of recursion was reached. In Figure 10, the parent-child relationships represented by solid arrows with full lines, the dependency relationships among explicit task instances represented by hollow arrows with dashed lines and the attached related information such as task type, execution time, etc, are all displayed in the graph. To simplify the discussion, we ignored the dependency relationships generated by implicit task instances and barrier directives. Different threads are described by different colors to be easily distinguished. The graph shows quite clearly the relationships of the task instances and their distribution among threads, which could easily identify the issue of load balance and find the performance spot.

## Conclusions

This paper introduces a monitoring mechanism and two demonstration graphs for task-based OpenMP paradigm and implements respective prototype modules to assist fast building performance tools. The key characteristics of our method are summarized as follows. First, simple as it is, the monitoring mechanism presented in this work has a greater generality and accessibility, independent of program, library source, or any special compiling or linking. Second, the monitoring library could acquire rich information with smaller overhead. Third, the demonstration graphs could reveal the task behavior from different perspectives, thus helping us identify performance problems.

In the future, more work can be done to perfect the mechanism with greater reliability and to extend the analysis graphs. Furthermore, the research of performance monitoring and analysis with task-based OpenMP paradigm based on other compiler implementations can be designed as well.
